# Quality of Life and Mental Health in Patients with Chronic Diseases Who Regularly Practice Yoga and Those Who Do Not: A Case-Control Study

**DOI:** 10.1155/2013/702914

**Published:** 2013-06-06

**Authors:** Holger Cramer, Romy Lauche, Jost Langhorst, Gustav Dobos, Anna Paul

**Affiliations:** Department of Internal and Integrative Medicine, Kliniken Essen-Mitte, Faculty of Medicine, University of Duisburg-Essen, Am Deimelsberg 34a, 45276 Essen, Germany

## Abstract

While clinical trials have shown evidence of efficacy of yoga in different chronic diseases, subjective health benefits associated with yoga practice under naturalistic conditions have not yet been investigated. The aim of this study was to investigate associations of regular yoga practice with quality of life and mental health in patients with chronic diseases. Using a case-control design, patients with chronic diseases who regularly practiced yoga were selected from a large observational study and compared to controls who did not regularly practice yoga and who were matched individually to each case on gender, main diagnosis, education, and age (within 5 years). Patients' quality of life (SF-36 questionnaire), mental health (Hospital Anxiety and Depression Scale), life satisfaction, and health satisfaction (Questionnaire for Life Satisfaction) were assessed. Patients who regularly practiced yoga (*n* = 186) had a better general health status (*P* = 0.012), a higher physical functioning (*P* = 0.001), and physical component score (*P* = 0.029) on the SF-36 than those who did not (*n* = 186). No group differences were found for the mental scales of the SF-36, anxiety, depression, life satisfaction, or health satisfaction. In conclusion, practicing yoga under naturalistic conditions seems to be associated with increased physical health but not mental health in chronically diseased patients.

## 1. Introduction

Yoga has its roots in Indian philosophy and has been a part of traditional Indian spiritual and medical practice for around 5000 years [1]. While the ultimate goal of traditional yoga has been described as uniting mind, body, and spirit, yoga has become a popular means to promote physical and mental well-being [1, 2]. As such, yoga has been adapted as part of complementary and alternative medicine in Western societies [3]. While yoga traditionally also comprises advice for ethical lifestyle and spiritual practice [1–4], it is most often associated with physical postures (asanas), breathing techniques (pranayama), and meditation (dhyana) in Western societies [2]. Different yoga schools have emerged that put varying focus on physical and mental practices [2]. However, even exercise-based yoga interventions differ from purely gymnastic exercises in that the yoga practitioner focuses his mind on the postures with inner awareness and a meditative focus of mind [5, 6]. 

Yoga is gaining increased popularity as a therapeutic practice. In 2008, about 15% of the American adult population was practicing yoga or was at least extremely interested in it [7]. Of those who were already practicing yoga, about half started practicing explicitly to improve their overall health, resulting in more than 13 million people practicing yoga for health reasons in 2007 [8, 9]. Worldwide, it is estimated that yoga is regularly practiced by about 30 million people [10].

Yoga has also been recognized as medical therapy; nearly 14 million Americans (6.1% of the population) reported that a physician or other therapist recommended yoga to them [7]. In the United Kingdom, yoga was even promoted by national health services as a safe and effective means to promote health in healthy and diseased individuals of all age groups [11]. 

Randomized-controlled trials and systematic reviews have investigated the efficacy of yoga in a number of physical conditions. It has been shown to improve health in patients with chronic low back pain [12], chronic neck pain [13], fibromyalgia [14], rheumatoid arthritis [15], osteoarthritis [15], cancer [16, 17], and menopausal symptoms [18]. It can reduce risk factors for cardiovascular disease [19] and improve risk profiles in adults with type 2 diabetes mellitus [20].

It has, however, been shown that self-administered yoga practice outside of yoga courses is important for sustained health benefits [6, 21]. Moreover, patients participating in a randomized-controlled trial might not be totally representative of the patient population [22]. Therefore, while clinical trials are important to establish evidence of efficacy of yoga in chronic diseases, studies on differences between patients who practice yoga (outside of clinical trials) and those who do not seem warranted. The aim of this study was to investigate differences in quality of life and mental health in patients with chronic diseases who regularly practice yoga and those who do not use a case-control design. It was hypothesized that patients who regularly practiced yoga had higher health-related quality of life and mental health than those who did not.

## 2. Materials and Methods

### 2.1. Patients

Patients were recruited from a large observational study that was conducted at a German Department for Internal and Integrative Medicine as part of its ongoing quality assurance program. All patients admitted to the Department during a 3-year period received detailed study information and were invited to participate in the study. Adults (aged 18 or older) with chronic diseases of rheumatological, gastrointestinal, pulmonological, and cardiovascular origin, including those with chronic pain syndromes, were eligible. Prior to inpatient treatment, patients who were willing to participate signed informed consent forms and completed questionnaires on complementary therapies use, health-related quality of life, mental health, life and health satisfaction [23].

This reanalysis used a case-control design. Patients who reported to engage in regular yoga practice (at least once weekly for at least 20 minutes) were regarded as cases. Matched pairs were created by randomly assigning a control subject from all possible controls, that is, patients who did not report to regularly practice yoga and who were exactly matched individually to each case on gender, main diagnosis, and education and as close as possible on age (within 5 years).

### 2.2. Questionnaires

#### 2.2.1. Yoga Practice and Sociodemographic Data

Patients were queried on whether they had ever practiced yoga before. Patients who reported prior yoga practice were further queried how often they were practicing yoga in a typical week (0 to 7 days per weeks) and whether they perceived yoga as helpful or harmful for their disease. Sociodemographic data on age, gender, family status, education, and employment were assessed. With respect to clinical data, ICD-10 diagnoses [24] were recorded.

#### 2.2.2. Health-Related Quality of Life

Health-related quality of life was assessed using the short-form 36 health survey questionnaire (SF-36) [25]. This instrument assesses health-related quality of life during the previous 4 weeks on eight subscales (physical functioning, physical role functioning, bodily pain, general health perceptions, vitality, social functioning, emotional role functioning, and mental health) and 2 main component summaries (mental component summary, physical component summary). Each scale can range from 0 to 100, with higher values indicating better quality of life. Patients rated their general health status on an unscaled item as “excellent,” “very good,” “good,” “fair” or “poor.” The SF-36 is the most commonly used instrument to assess health-related quality of life in patients with chronic diseases and has proven excellent validity and reliability [25].

#### 2.2.3. Mental Health

Mental health was assessed using the Hospital Anxiety and Depression Scale (HADS). This instrument has 14 items, scored on 4-point Likert scales [26]. Higher scores indicate more severe symptoms. For both dimensions, cut-off scores have been established that indicate possible subsyndromal (≥8) or clinically relevant (≥11) anxiety or depression [27]. The HADS has been specifically developed as an instrument to detect anxiety and depression in patients with physical conditions. Reliance on aspects of mental conditions that are also symptoms of physical diseases, for example, fatigue or sleep disorders, was avoided [26]. The instrument has excellent validity and reliability [27].

#### 2.2.4. Life Satisfaction and Health Satisfaction

Life satisfaction and health satisfaction were assessed using a 5-point Likert scale item, each from the questionnaire for life satisfaction (FLZ) [28]. Life satisfaction was queried as follows “Considering your current situation, how satisfied are you with your overall life? and satisfaction with health as all in all, how satisfied are you with your health?” The endpoints ranged from 1 = very unsatisfied and 5 = very satisfied.

### 2.3. Statistical Analysis

Sociodemographic and clinical data were analyzed descriptively. Success of matching was tested by comparing sociodemographic and clinical data between groups. For this purpose and to identify differences between groups in health-related quality of life, mental health, and satisfaction, tests for matched pairs were used. Paired *t*-tests were used for interval-scaled data, McNemar's test for nominal data, and the sign test for ordinal data [29]. A *P* value of ≤0.05 was considered statistically significant for all analyses. Analyses were conducted using SPSS (release 20.0, IBM, Armonk, NY, USA).

## 3. Results

### 3.1. Patients' Characteristics

During the study period, 2804 patients were admitted to the hospital. Of those, 2486 (88.7%) agreed to participate in the study. One hundred and eighty-six patients reported to engage in regular yoga practice and were matched to 186 control subjects. Of patients who regularly practiced yoga, 129 reported health benefits and 3 reported negative effects associated with yoga practice.

Patients' characteristics are shown in Table 1. The study sample mainly consisted of women in their 50s; about a third of the patients had been educated to A-level standard; and about half of the patients were in a relationship and employed. More than two-thirds of the patients experienced a chronic pain condition, with back pain, headache, fibromyalgia, and rheumatic arthritis being the most frequently causes for admission. The case and the control groups did not differ significantly on any of these variables.

### 3.2. Health-Related Quality of Life, Mental Health, Life Satisfaction, and Health Satisfaction

**Figure 1:**
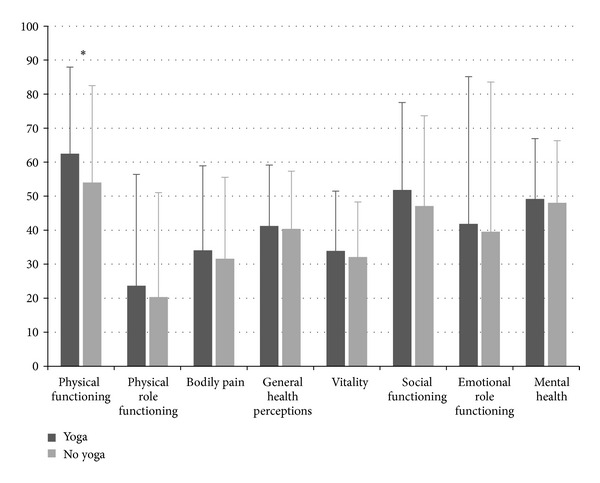
Health-related quality of life (SF-36 subscales) in patients with chronic diseases who regularly practice yoga and those who do not (mean + standard deviation). Asterisks indicate significant group differences.

Patients who regularly practiced yoga reported a better general health status with more patients describing their health status as “excellent,” “good,” or “fair,” and less patients describing it as “poor” compared to those who did not regularly practice yoga (Table 2). Yoga practicing patients also reported a higher physical component summary for health-related quality of life (Table 3). Scores on all SF-36 subscales were higher in cases than in controls with only the physical functioning subscale showing significant group differences (*P* = 0.001) (Figure 1). No significant group differences were found for anxiety, depression, life satisfaction, or health satisfaction (Table 3).

## 4. Discussion

Using a case-control design, this study compared 186 patients with chronic diseases who regularly practiced yoga with 186 matched controls who did not. As hypothesized, yoga practitioners had better general health status and physical quality of life. In contrast, mental quality of life, mental health, life satisfaction, and health satisfaction did not differ significantly between groups. However, even while not reaching statistical significance, yoga practitioners scored higher on all dimensions of quality of life in the SF-36 including mental quality of life.

To the best of our knowledge, this is the first case-control study investigating subjective health benefits associated with yoga practice under naturalistic conditions. Prior case-control studies have mainly investigated differences between healthy people or samples drawn from the general population on anatomical or physiological parameters. In a small case-control study, Chaya et al. [30] found increased insulin sensitivity in yoga practitioners compared to nonusers. Two Chinese studies compared magnetic resonance images in healthy yoga practitioners and non-practitioners and found increased risk of meniscus injuries [31] but decreased risk of degenerative disc disease in yoga practitioners [32]. A recent case-control study investigated brain-grounded maps of the body using a motor imagery task and found no differences between yoga practitioners and matched controls [33]. However, an uncontrolled cross-sectional study found linear associations between frequency of yoga practice under naturalistic conditions and subjective measures of health [34]. None of these studies involved patients with chronic diseases.

Effects of yoga on patients with chronic diseases have been investigated in a number of randomized-controlled trials. A recent meta-analysis found strong evidence for short-term effectiveness and moderate evidence for long-term effectiveness of yoga for chronic low back pain in the most important patient-centered outcomes [12]. Similarly, evidence of effectiveness has been reported by meta-analyses on yoga for fibromyalgia [14], other pain [35], and fatigue [36]. Yoga has also been reported to be effective in improving health status and quality of life in patients with arthritis [15], cardiovascular conditions [19, 37], and lung diseases [37]. In contrast to the findings of the present studies, yoga has also been shown to improve mental health in patients with psychological disorders [37–40] and physical conditions [14, 17, 18, 35]. Patients in both groups of this study reported high levels of depression and anxiety that reached borderline levels of generalized anxiety disorder [27]. The lack of significant group differences in measures of mental health suggests that the positive short-term effects that were found in clinical trials [14, 17, 18, 35, 37–41] might not be there under naturalistic conditions and/or not persist with sustained yoga practice.

How might yoga improve health status in patients with chronic diseases? Regarding chronic pain, the isometric exercises practiced during yoga have been shown to relieve pain and muscle spasm [13]. Moreover, yoga puts a focus on increasing awareness of muscle tonus and joint position and is also thought to help recognizing and changing habitual patterns of posture and muscle tension in daily life [6, 13]. Yoga has been shown to reduce stress [42], body weight, blood pressure, and hyperlipidemia [19, 43], conditions that are associated with chronic diseases such as coronary heart disease and diabetes [44]. Practice of yoga posture but not of yogic meditation has been shown to improve lung function and cardiovascular capacity. Thereby, yoga can improve overall fitness and exercise performance [37].

Three patients (1.6%) reported negative effects associated with their yoga practice. While positive effects of yoga are extensively researched, data on incidence rates of negative effects and adverse events are rare. A Finnish survey found that 62% of 110 Ashtanga Vinyasa Yoga practitioners reported at least 1 yoga-related musculoskeletal injury [45]. More recently, in a large national survey, 21.3% of about 2500 Australian yoga practitioners indicated that they had been injured during yoga in the past [46]. Therefore, the incidence rate of 1.3% in this analysis might underestimate the actual risks of yoga practice.

What is the specifically new this study adds? This study shows for the first time that practicing yoga under naturalistic conditions is associated with better general health status and physical quality of life in patients with chronic diseases. Moreover, yoga practice seems to be generally safe for this patient population. Yoga practice might, therefore, be recommended to patients with a variety of chronic diseases to improve their overall health and physical wellbeing.

The study reported here has several limitations. First, while cases and controls were matched on the most important sociodemographic and clinical variables, they might, however, still differ on some undetected parameters that enhance both yoga practice and health status. In this case, the association between yoga practice and health status might be a mere statistical artifact. Second, frequency of yoga practice, length of practice, and the specific yoga school where patients were engaged in were not assessed. Third, the study design did not control for total exercise time of cases and controls. Therefore, the differences between patients who regularly practiced yoga and those who did not reported here cannot be regarded as a necessarily specific effect of yoga practice but can also be attributed to the physical activity associated with yoga. The contribution of different components of yoga such as physical postures, breathing techniques, or meditation to its health-promoting effect is still an object of investigation.

Future research should address these limitations. Specifically, studies should investigate differences between different yoga schools and yoga practices, such as physical postures, breathing techniques, and meditation. Each of these practices has been shown to be associated with effects on specific health variables in an uncontrolled cross-sectional study [34]. To define the optimal dose of yoga practice, subgroups of yoga practitioners with different practice intensity should be compared. The role of physical activity in the health benefits of yoga could be investigated by controlling for total exercise time. Finally, the analysis presented here is limited to patients with internal diseases. Future studies could replicate this analysis in other patient groups such as patients with mental diseases.

In conclusion, patients with chronic diseases who regularly practiced yoga reported better overall health status and physical quality of life than those who did not. Practicing yoga under naturalistic conditions seems to be associated with improved physical health in chronically diseased patients.

## Figures and Tables

**Table 1:** Sociodemographic and clinical characteristics of patients with chronic diseases who practice yoga and those who do not (mean ± standard deviation).

	Yoga (*n* = 186)	No yoga (*n* = 186)	*P*
Age	51.8 ± 12.8	51.5 ± 12.9	0.151
Gender *n* (%)			1.000
Female	165 (88.7%)	165 (88.7%)	
Male	21 (11.3%)	21 (11.3%)	
Education *n* (%) with A-level and higher	66 (35.5%)	66 (35.5%)	1.000
Family status *n* (%) in relationship/married	105 (56.5%)	110 (59.1%)	0.630
Employment *n* (%)			0.823
Unemployed	103 (55.4%)	100 (53.8%)	
Part-time employed	33 (17.7%)	33 (17.7%)	
Full-time employed	50 (26.9%)	53 (28.5%)	
Diagnosis *n* (%)			1.000
Spinal pain	34 (18.3%)	34 (18.3%)	
Osteoarthritis	16 (8.3%)	16 (8.3%)	
Rheumatic arthritis	9 (4.8%)	9 (4.8%)	
Fibromyalgia	27 (15.55)	27 (15.55)	
Headache	20 (10.8%)	20 (10.8%)	
Pain, others	18 (9.7%)	18 (9.7%)	
Hypertension	7 (3.8%)	7 (3.8%)	
Ischemic cardiac disease	2 (1.1%)	2 (1.1%)	
Inflamm. bowel disease	14 (7.5%)	14 (7.5%)	
Irritable bowel syndrome	9 (4.8%)	9 (4.8%)	
Lung diseases	8 (4.3%)	8 (4.3%)	
Others	22 (1.8%)	22 (1.8%)	

**Table 2:** General health status of patients with chronic diseases who practice yoga and those who do not.

	Yoga (*n* = 186)	No yoga (*n* = 186)	*P*
General health status *n* (%)			0.012
Excellent	1 (0.5%)	0 (0.0%)	
Very good	2 (1.1%)	2 (1.1%)	
Good	32 (17.2%)	26 (14.0%)	
Fair	116 (62.4%)	101 (54.3%)	
Poor	35 (18.8%)	57 (30.6%)	

**Table 3:** Health-related quality of life, mental health, and satisfaction in patients with chronic diseases who practice yoga and those who do not (Mean ± SD).

	Yoga	No yoga	Group difference (95% CI)	*P*
Health-related quality of life (SF-36)				
Physical component score	35.7 ± 9.1	33.7 ± 9.9	2.0 (0.2; 3.7)	0.029
Mental component score	37.3 ± 11.6	36.9 ± 11.5	0.5 (−2.2; 3.2)	0.737
Anxiety	9.9 ± 4.0	9.6 ± 3.9	0.3 (−0.6; 1.1)	0.518
Depression	7.5 ± 3.5	7.7 ± 4.0	−0.2 (−1.0; 0.5)	0.563
Life satisfaction	3.4 ± 0.9	3.3 ± 1.0	0.1 (−0.1; 0.3)	0.252
Health satisfaction	2.2 ± 1.0	2.1 ± 1.0	0.1 (−0.1; 0.3)	0.165
